# Circular RNA hsa_circ_0083756 promotes intervertebral disc degeneration by sponging miR‐558 and regulating TREM1 expression

**DOI:** 10.1111/cpr.13205

**Published:** 2022-02-21

**Authors:** Xianfa Du, Shunlun Chen, Haitao Cui, Yuming Huang, Jianru Wang, Hui Liu, Zemin Li, Chunxiang Liang, Zhaomin Zheng, Hua Wang

**Affiliations:** ^1^ Department of Spine Surgery Guangdong Provincial Key Laboratory of Orthopaedics and Traumatology The First Affiliated Hospital of Sun Yat‐sen University Guangzhou China; ^2^ Department of Orthopedics The Affiliated Hospital of Qingdao University Qingdao China; ^3^ Pain Research Center Sun Yat‐sen University Guangzhou China

**Keywords:** circular RNA, hsa_circ_0083756, IVDD, low back pain, miR‐558, TREM1

## Abstract

**Objectives:**

Intervertebral disc degeneration (IVDD) is a leading cause of low back pain. Circular RNAs (circRNAs) have been demonstrated to exert vital functions in IVDD. However, the role and mechanism of hsa_circ_0083756 in the development of IVDD remain unclear.

**Materials and methods:**

RT‐qPCR was performed to detect expressions of hsa_circ_0083756, miR‐558 and TREM1 in nucleus pulposus (NP) tissues and cells. CCK8 assay, flow cytometry, TUNEL assay, RT‐qPCR and WB were used to clarify the roles of hsa_circ_0083756 in NP cells proliferation and extracellular matrix (ECM) formation. Bioinformatics analyses, dual‐luciferase reporter gene experiment, RNA immunoprecipitation (RIP) assay and FISH assay were performed to predict and verify the targeting relationship between hsa_circ_0083756 and miR‐558, as well as that between miR‐558 and TREM1. Ultimately, the effect of hsa_circ_0083756 on IVDD was tested through anterior disc‐puncture IVDD animal model in rats.

**Results:**

hsa_circ_0083756 was upregulated in degenerative NP tissues and cells. *In vitro* loss‐of‐function and gain‐of‐function studies suggested that hsa_circ_0083756 knockdown promoted, whereas hsa_circ_0083756 overexpression inhibited NP cells proliferation and ECM formation. Mechanistically, hsa_circ_0083756 acted as a sponge of miR‐558 and subsequently promoted the expression of TREM1. Furthermore, *in vivo* study indicated that silencing of hsa_circ_0083756 could alleviate IVDD in rats.

**Conclusions:**

hsa_circ_0083756 promoted IVDD via targeting the miR‐558/TREM1 axis, and hsa_circ_0083756 may serve as a potential therapeutic target for the treatment of IVDD.

## Graphical abstract

1. hsa_circ_0083756 is generated from the back‐spliced exon 3 of TRIM35. 2. hsa_circ_0083756 serves as a sponge for miR‐558 in NP cells. 3. TREM1 is the target gene of miR‐558. 4. hsa_circ_0083756 regulates IVDD by modulating the miR‐558/TREM1 axis.

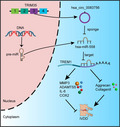

## 1 INTRODUCTION

Low back pain (LBP) is a major contributor to disability and causes great economic and social burdens around the world^1^, ^2^; and intervertebral disc degeneration (IVDD) is the predominant cause of LBP.^3^, ^4^ The pathological hallmarks of IVDD are decreased nucleus pulposus (NP) cells and degradation of the extracellular matrix (ECM) caused by increased matrix‐degrading enzymes.^5^, ^6^ Although biological knowledge is rising, and numerous basic studies on IVDD have been conducted, the treatment for IVDD remains unsatisfactory, suggesting that the disorder is still poorly understood and difficult to cure.^7^, ^8^ This highlights the need for further investigation of the mechanisms and innovative therapies for IVDD.

Circular RNAs (circRNAs) represent a novel subtype of endogenous non‐coding RNA with covalently closed loop structures lacking 5′‐3′ ends and a polyadenine tail.^9^ Mounting evidence shows that circRNAs were involved in multiple vital biological processes, including cell metabolism, proliferation, differentiation and survival.^10^, ^11^, ^12^, ^13^, ^14^ In addition, circRNAs' expression patterns are closely related to various diseases, such as cardiovascular disease,^15^ neurological disease,^16^ acute myeloid leukaemia,^17^ human cancers^18^ and osteoarthritis.^19^ Furthermore, multiple studies have confirmed that circRNAs are rich in micro RNA (miRNA) binding sites and exert their critical functions by sponging miRNAs.^20^ For example, Wu et al. found that circPDE4D exerts its function of protecting against osteoarthritis by functioning as a sponge of miR‐103a‐3p.^19^ Consistently, circ‐KRT6C promoted malignant progression and immune evasion of colorectal cancer via acting as a miR‐485‐3p sponge.^21^ Recently, increasing evidence has revealed that many circRNAs are also differently expressed between non‐degenerated and degenerated NP tissues,^22^ indicating their potential functions in the development of IVDD. Although some circRNAs have been revealed diverse functions in the regulation of IVDD,^23^ the role and underlying mechanisms of circRNAs in IVDD largely remain vague and need further investigation.

In this study, we performed a comprehensive bioinformatic analysis based on our previous circRNA array data^24^ and circRNA data set (GSE67566) downloaded from the Gene Expression Omnibus (GEO) database.^25^, ^26^ As a result, we identified several IVDD‐specific circRNAs and revealed that hsa_circ_0083756 was significantly increased in degenerated NP tissues compared with non‐degenerated NP tissues. Our *in silico* analysis led us to predict that hsa_circ_0083756 had potential binding sites of miR‐558, which validated the correlation between hsa_circ_0083756 and miR‐558 in NP cells. Further, we predicted that TREM1 was the target gene of miR‐558. Subsequently, gain and loss of function were studied *in vitro* and *in vivo* experiments to systematically clarify its role in the pathogenesis mechanism of IVDD and identify its therapeutic potential.

## 2 RESULTS

### 2.1 Identification of differentially expressed circRNAs

**FIGURE 1:**
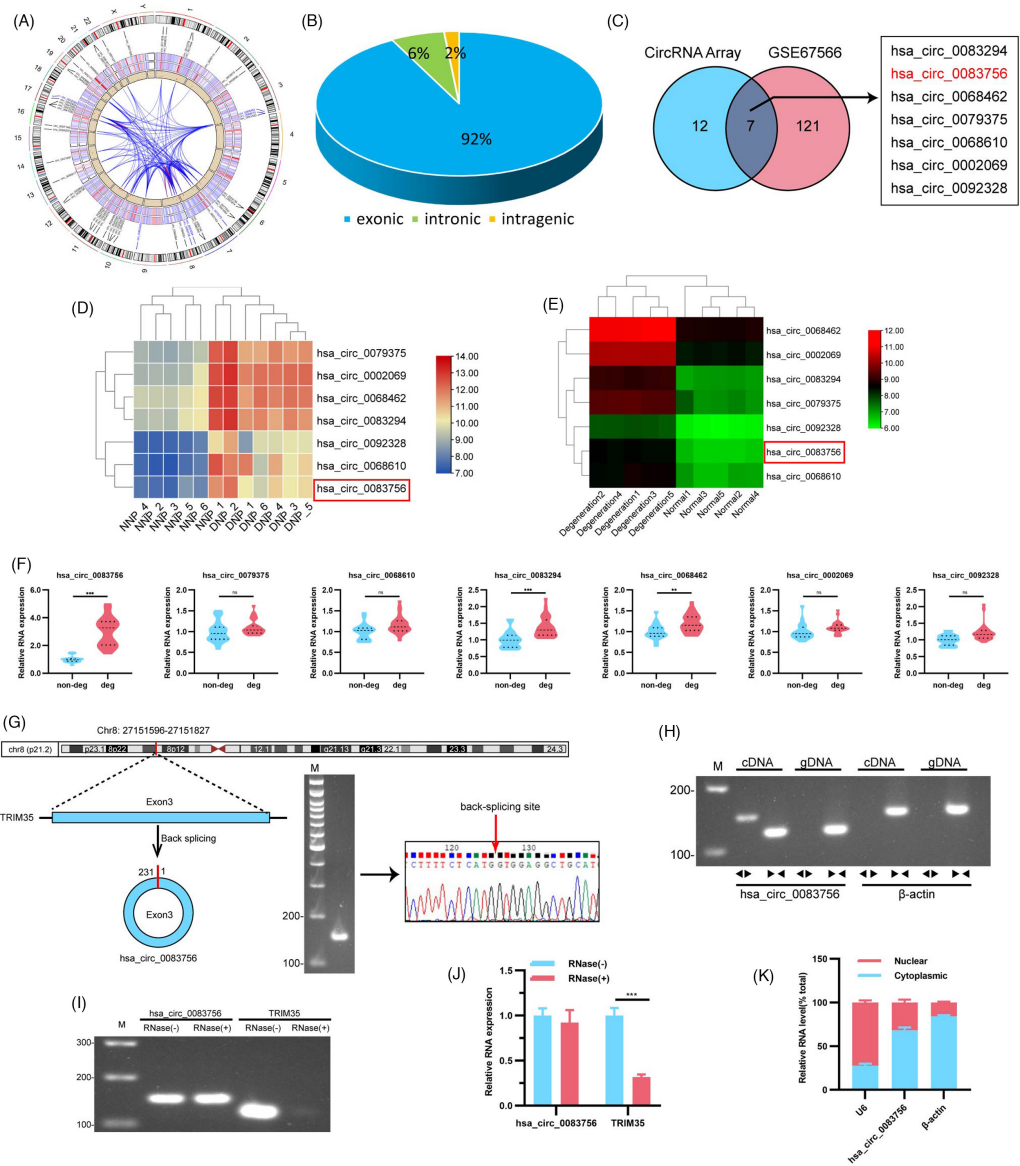
Expression profiles of circRNAs in non‐degenerated and degenerated NP tissues. (A) Circos plot displayed the distribution and expression of circRNAs on human chromosomes in our circRNA Array. The outermost layer was a chromosome map of the human genome. The inner circles from outside to inside corresponded to differentially expressed circRNAs, a heatmap of all circRNAs, a scatter plot and a cross‐link of differentially expressed circRNAs. (B) Constituent ratios of differentially expressed circRNAs. (C) Venn diagram demonstrating the overlap of circRNAs in the circRNA Array (right) and GSE67566 (left). (D, E) Hierarchical cluster analysis of the seven circRNAs in the circRNA Array and GSE67566 data sets. (F) Expression of the seven cirRNAs in non‐degenerated and degenerated NP tissues was measured using RT‐qPCR; *n* = 10 for non‐degenerated NP tissues and *n* = 14 for degenerated NP tissues. (G) Schematic illustration demonstrating the circularization of TRIM35 exon 3 to form circ‐83756. The presence of circ‐83756 was validated by RT‐PCR, followed by agarose gel electrophoresis and sanger sequencing. The red arrow represents the head‐to‐tail splicing site of circ‐83756. (H) RT‐PCR validated the existence of circ‐83756, which was amplified by divergent primers in cDNA but not gDNA; β‐actin was used as control. (I, J) The expressions of circ‐83756 and TRIM35 mRNA were detected by RT‐qPCR in the presence or absence of RNase R. (K) Expression levels of cytoplasmic control transcripts (β‐actin), the nuclear control transcript (U6) and circ‐83756 were determined by RT‐qPCR in the cytoplasmic and nuclear fractions of NP cells. **p < 0.01 and ***p < 0.001 vs. the indicated group; ns: no significant; statistical data were presented as mean ±SEM; IVDD, intervertebral disc degeneration

Our previous study identified differentially expressed circRNAs between non‐degenerated and degenerated IVD NP tissues.^24^ The Circos plot showed that the differentially expressed circRNAs came from all chromosomes (Figure 1A). In addition, amongst them, 92% of the differentially expressed circRNAs were transcribed from protein‐coding exons, 6% were from introns, and 2% were from intragenic regions (Figure 1B). We overlapped microarray analysis of our previous data and microarray data set (GSE67566) acquired from the Gene Expression Omnibus (GEO) database^25^, ^26^ to further screen common deferentially expressed circRNAs. The thresholds were set as follows: fold changes >3 and *p*‐values <0.05 as determined by the Student's *t*‐test. As a result, seven circRNAs upregulated in IVDD were obtained at the intersection (Figure 1C). Hierarchical clustering showed that the expression patterns of these seven circRNAs were distinguishable between non‐degenerated and degenerated IVD NP tissues in both our previous data and the GSE67566 data set (Figure 1D,E). Further, RT‐qPCR was used to verify the expressions of these seven circRNAs in degenerated IVD specimens compared with non‐degenerated IVD specimens. The results showed that hsa_circ_0083756 had the highest increase; therefore, we chose hsa_circ_0083756 (circ‐83756) for further study (Figure 1F). In the subsequent steps, the characteristics of circ‐83756 were explored. Circ‐83756 is generated from the back‐spliced exon 3 of TRIM35, which is located on chromosome 8p21.2 (chr8:27151596–27151827). Next, we designed divergent primers for the specific back‐splicing sites. RT‐PCR and agarose gel electrophoresis were used to validate the efficacy and specificity of the divergent primers. As shown in Figure 1G, there was only a single distinct band of the expected PCR product size, indicating that there were no primer dimers or nonspecific amplification. Further, the head‐to‐tail splicing in the RT‐PCR product was confirmed using sanger sequencing, suggesting the presence of the circular junction (Figure 1G).

Convergent primers and specific divergent primers were designed to amplify TRIM35 mRNA and circ‐83756. Genomic DNA (gDNA) and transcriptomic complement DNA (cDNA) were obtained from NP cells and subsequently applied as templates for RT‐PCR. Notably, convergent primers could amplify TRIM35 mRNA in both the cDNA and gDNA templates, whereas circ‐83756 could be amplified by the divergent primer from cDNA but not from gDNA (Figure 1H). Furthermore, RNase R exonuclease digestion was used to validate whether circ‐83756 exhibits high stability. The agarose gel electrophoresis and RT‐qPCR results showed that most of the linear TRIM35 mRNA was digested by RNase R but circ‐83756 was resistant to RNase R (Figure 1I,J). Moreover, RT‐qPCR analysis of the nuclear and cytoplasmic fractions of RNA revealed that circ‐83756 was primarily localized in the cytoplasm (Figure 1K).

### 2.2 Roles of circ‐83756 in the proliferation, apoptosis, ECM synthesis and degradation, and inflammatory factor release in NP cells

**FIGURE 2:**
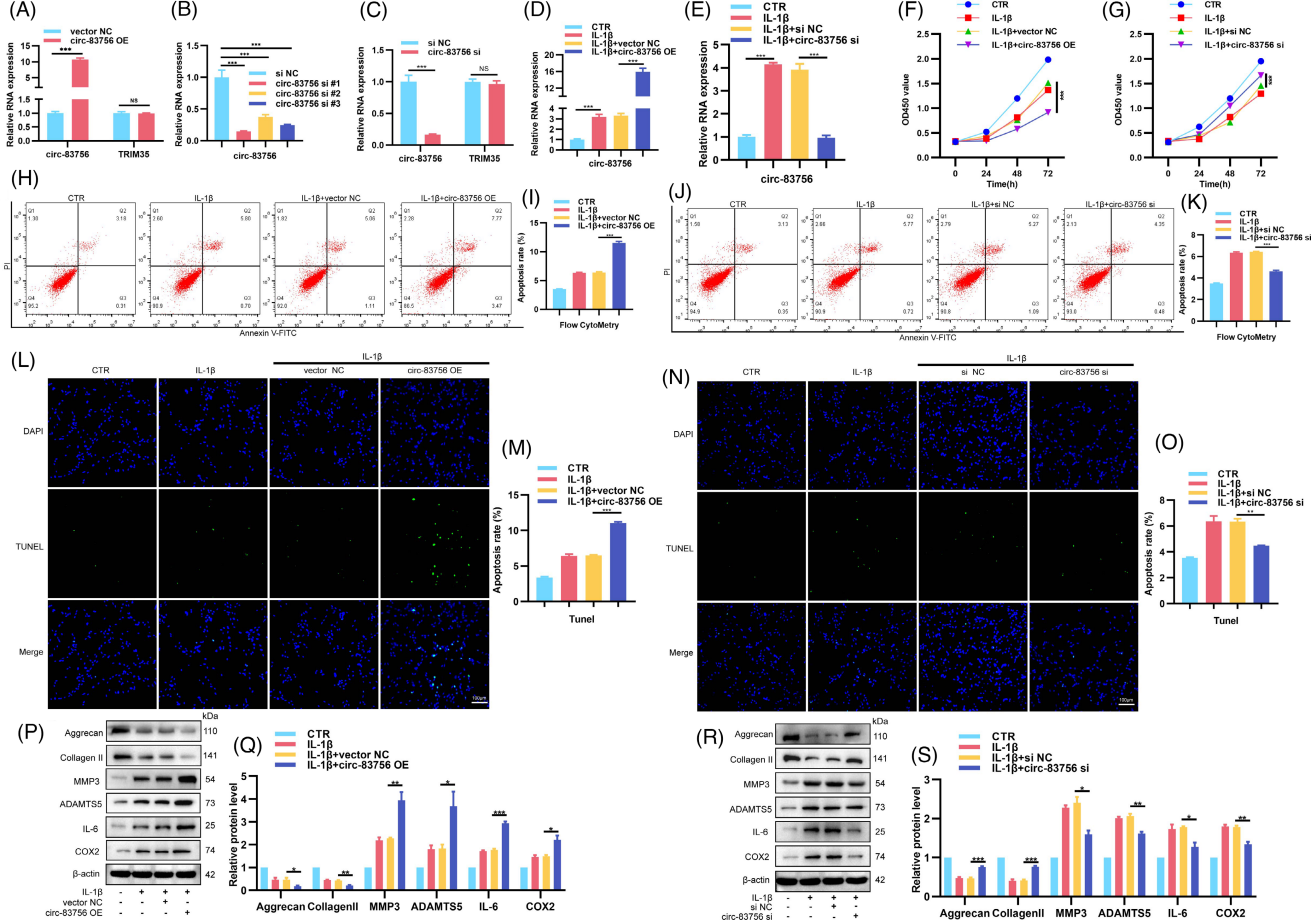
Role of circ‐83756 in the proliferation, apoptosis, ECM synthesis and degradation, and inflammatory factors release in NP cells. (A–C) NP cells transfected with circ‐83756 OE plasmid or three different circ‐83756 siRNAs or their negative control; the expressions of circ‐83756 and linear TRIM35 were evaluated using RT‐qPCR. (D, E) IL‐1β increased the expression of circ‐83756 in NP cells; circ‐83756 OE enhanced and circ‐83756 si suppressed the expression of circ‐83756. (F, G) NP cells were treated by IL‐1β with or without circ‐83756 OE or circ‐83756 si, and NP cell proliferation was examined by the CCK8 assay. (H–O) The effect of circ‐83756 OE or circ‐83756 si on NP cell apoptosis was detected by flow cytometry (H–K) and TUNEL assay (L–O). (P–S) The protein levels of ECM and inflammatory factor‐related genes (aggrecan, collagen Ⅱ, MMP3, ADAMTS5, IL‐6 and COX2) were measured via western blot analysis. *p < 0.05, **p < 0.01 and ***p < 0.001 vs. the indicated group. Statistical data were presented as mean ±SEM; FITC, fluorescein isothiocyanate; PI, propidium iodide; TUNEL, terminal dexynucleotidyl transferase (TdT)‐mediated dUTP nick‐end labelling; DAPI, 4′,6‐diamidino‐2‐phenylindole; siRNA, small interfering RNA

To explore the role of circ‐83756 in the proliferation, apoptosis, ECM synthesis and degradation, and inflammatory factor release in NP cells, we performed gain and loss‐of‐function experiments. First, we constructed a circ‐83756 overexpression plasmid (circ‐83756 OE) and validated its efficiency via RT‐qPCR. As shown in Figure 2A, compared with its negative control, transfection with circ‐83756 OE significantly improved the expression of circ‐83756 in human NP cells but had no effect on the expression of linear TRIM35 mRNA. Then, we designed three small interfering RNAs (siRNAs) targeting the back‐splicing sites of circ‐83756. The RT‐qPCR results showed that circ‐83756 si #1 had the highest knockdown efficiency amongst the three siRNAs (Figure 2B). Therefore, circ‐83756 si #1 was chosen for further experiments. As circ‐83756 and linear TRIM35 share a partial sequence, we also tested the effects of circ‐83756 si on linear TRIM35 mRNA, and the RT‐qPCR results showed that circ‐83756 si inhibited the expression of circ‐83756 rather than linear TRIM35 mRNA (Figure 2C). Further, the overexpression effect of circ‐83756 OE and the silence effect of circ‐83756 si were also confirmed in the interleukin (IL)‐1β‐induced cellular model of IVDD (Figure 2D,E). CCK8 tests were used to detect NP cell proliferation. The results revealed that circ‐83756 OE significantly accelerated whilst circ‐83756 si partially ameliorated the inhibition of NP cell proliferation induced by IL‐1β (Figure 2F,G). Flow cytometry analysis with Annexin V–fluorescein isothiocyanate (FITC)/propidium iodide (PI) dual staining indicated that circ‐83756 OE clearly enhanced the rate of apoptosis in NP cells induced by IL‐1β. Conversely, circ‐83756 silencing remarkably suppressed the apoptotic effects caused by IL‐1β (Figure 2H–K). Transferase dUTP nick‐end labelling (TUNEL) staining further demonstrated that circ‐83756 OE significantly deteriorated the apoptotic effects caused by IL‐1β, whereas circ‐83756 silencing partly reversed the apoptosis rate of NP cells induced by IL‐1β (Figure 2L–O). The effects of circ‐83756 on the ECM synthesis and degradation of NP cells were investigated using western blot (WB). The results showed that circ‐83756 OE significantly enhanced the upregulation of MMP3, ADAMTS5, IL‐6 and COX2 and the downregulation of aggrecan and collagen Ⅱ induced by IL‐1β. In contrast, circ‐83756 silencing partially reversed the effects of IL‐1β on the expressions of these genes (Figure 2P–S). Overall, the overexpression and silencing of circ‐83756 regulated the proliferation, apoptosis, ECM synthesis and degradation, and inflammatory factors release in NP cells, which suggested the regulatory function of circ‐83756 on NP cells' biological processes.

### 2.3 Circ‐83756 served as a sponge for miR‐558 in NP cells

**FIGURE 3:**
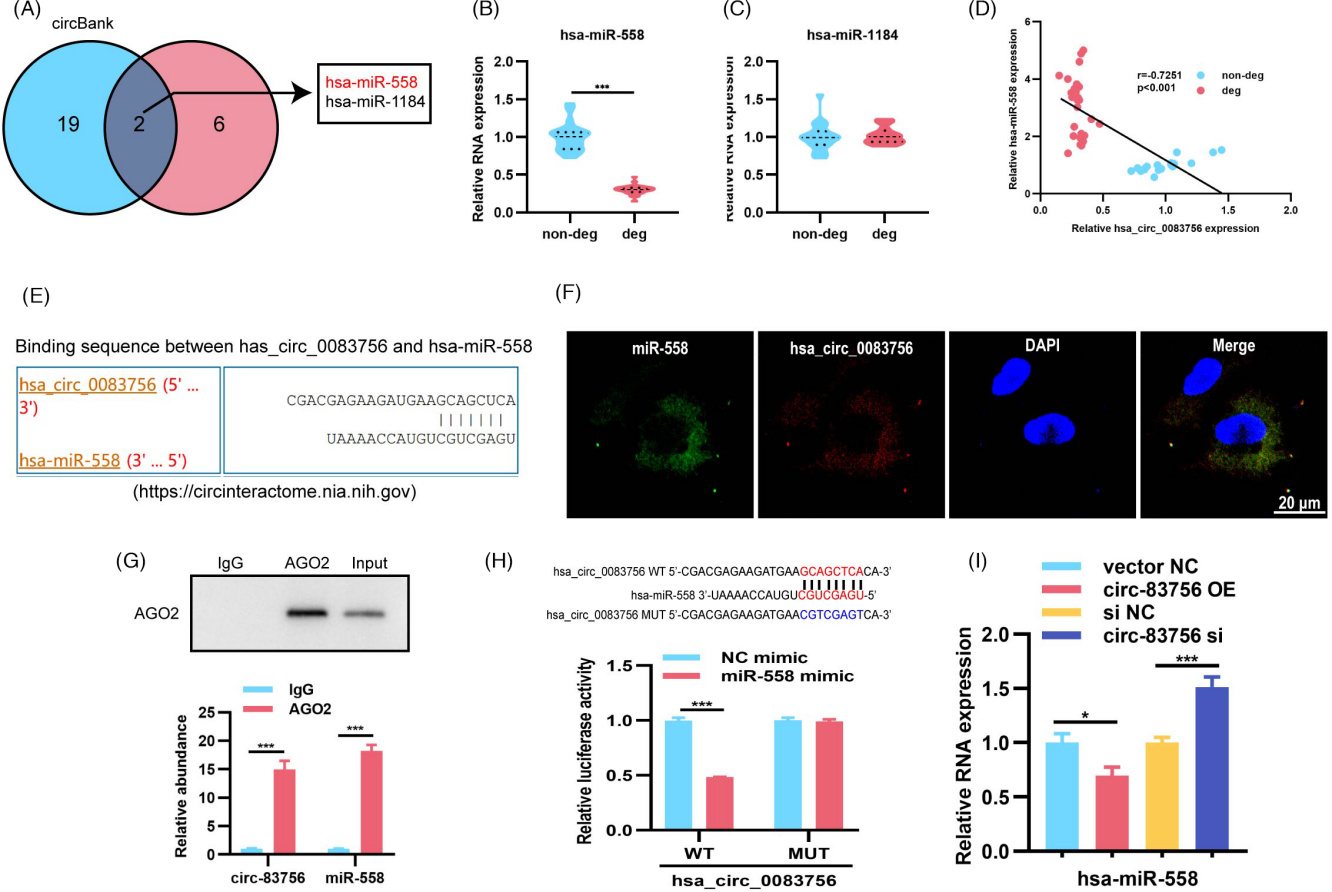
circ‐83756 acted as a sponge of miR‐558. (A) Venn diagram demonstrating the overlap of the predicted potential target miRNAs of circ‐83756 using the circBank and CircInteractome databases. (B, C) RT‐qPCR demonstrated that the expression of miR‐558 decreased in degenerated NP tissues, whilst miR‐1184 showed no significant difference; *n* = 10 for non‐degenerated NP tissues and *n* = 14 for degenerated NP tissues. (D) The expression of miR‐558 was significantly negatively correlated with circ‐83756 expression. (E) Schematic of the predicted miR‐558 sites in the circ‐83756 from CircInteractome online database. (F) RNA FISH images showing the colocalization of circ‐83756 and miR‐558 in NP cells, nuclei were stained with DAPI, green fluorescence indicated miR‐558, and red fluorescence indicated circ‐83756. (G) RNA immunoprecipitation confirmed that anti‐AGO2 antibodies immunoprecipitated circ‐83756 and miR‐558. AGO2 was detected using IP‐western blot (up panel), and circ‐83756 and miR‐558 expression levels were detected using qRT‐PCR (down panel). (H) Luciferase reporter assay showed the luciferase activities of Luc‐circ‐83756 WT and Luc‐circ‐83756 MUT in T293 cells co‐transfected with miR‐NC or miR‐558 mimic. (I) RT‐qPCR showed the expression of miR‐558 in NP cells treated with circ‐83756 OE or circ‐83756 siRNA. *p < 0.05 and ***p < 0.001 vs. the indicated group. Statistical data were presented as mean ±SEM; IVDD, intervertebral disc degeneration; FISH, fluorescence in situ hybridization; DAPI, 4′,6‐diamidino‐2‐phenylindole; WT, wild type; MUT, mutant type

As circRNAs have been illustrated to serve as miRNA sponges and circ‐83756 is predominantly located in the cytoplasm, we speculated that circ‐83756 could also sponge a specific miRNA and regulate its downstream functions. Here, circBank and CircInteractome interactions were used to predict the potential interacting miRNAs. The results showed that miR‐1184 and miR‐558 were the candidate miRNAs targeting circ‐83756 (Figure 3A). The expression patterns of miR‐1184 and miR‐558 in degenerated and non‐degenerated IVD NP tissues were detected by RT‐qPCR, which showed that miR‐558 expression was significantly downregulated in degenerated NP tissues (Figure 3B,C). Pearson correlation analysis showed that the expression of miR‐558 in NP tissues exhibited a negative correlation with the expression of circ‐8376 in NP tissues (Figure 3D). In addition, a previous study showed that miR‐558 could regulate the IL‐1β‐mediated induction of COX2 and catabolic effects in human articular chondrocytes,^27^ so miR‐558 was selected for further analysis.

Next, we investigated the ability of circ‐83756 to bind miR‐558. First, a search in the Circular RNA Interactome database revealed that there were potential binding sites between circ‐83756 and the miR‐558 seed region (Figure 3E). Fluorescent in situ hybridization (FISH) assay showed that circ‐83756 and miR‐558 were mainly expressed and colocalized in the cytoplasm (Figure 3F). Second, AGO2 RIP in human NP cells revealed that circ‐83756 and miR‐558 were both significantly enriched by anti‐AGO2 antibodies, indicating that they both existed in RNA‐induced silencing complex (RISC) (Figure 3G). Third, the binding sites of miR‐558 to circ‐83756 were directly validated via a dual‐luciferase reporter gene assay. We observed that the luciferase activity of the pmirGLO‐circ‐83756 wild‐type (WT) plasmid was significantly decreased after miR‐558 mimic transfection, whereas the luciferase activity of the pmirGLO‐circ‐83756 mutant‐type (MUT) plasmid containing mutated miR‐558 binding sites was not noticeably affected (Figure 3H). Furthermore, RT‐qPCR results demonstrated that the expression of miR‐558 was decreased by the circ‐83756 OE, whilst increased following the circ‐83756 silencing (Figure 3I). Taken together, these results suggested that circ‐83756 can bind to miR‐558 directly in NP cells.

### 2.4 Roles of miR‐558 in the proliferation, apoptosis, ECM synthesis and degradation, and inflammatory factor release in NP cells

**FIGURE 4:**
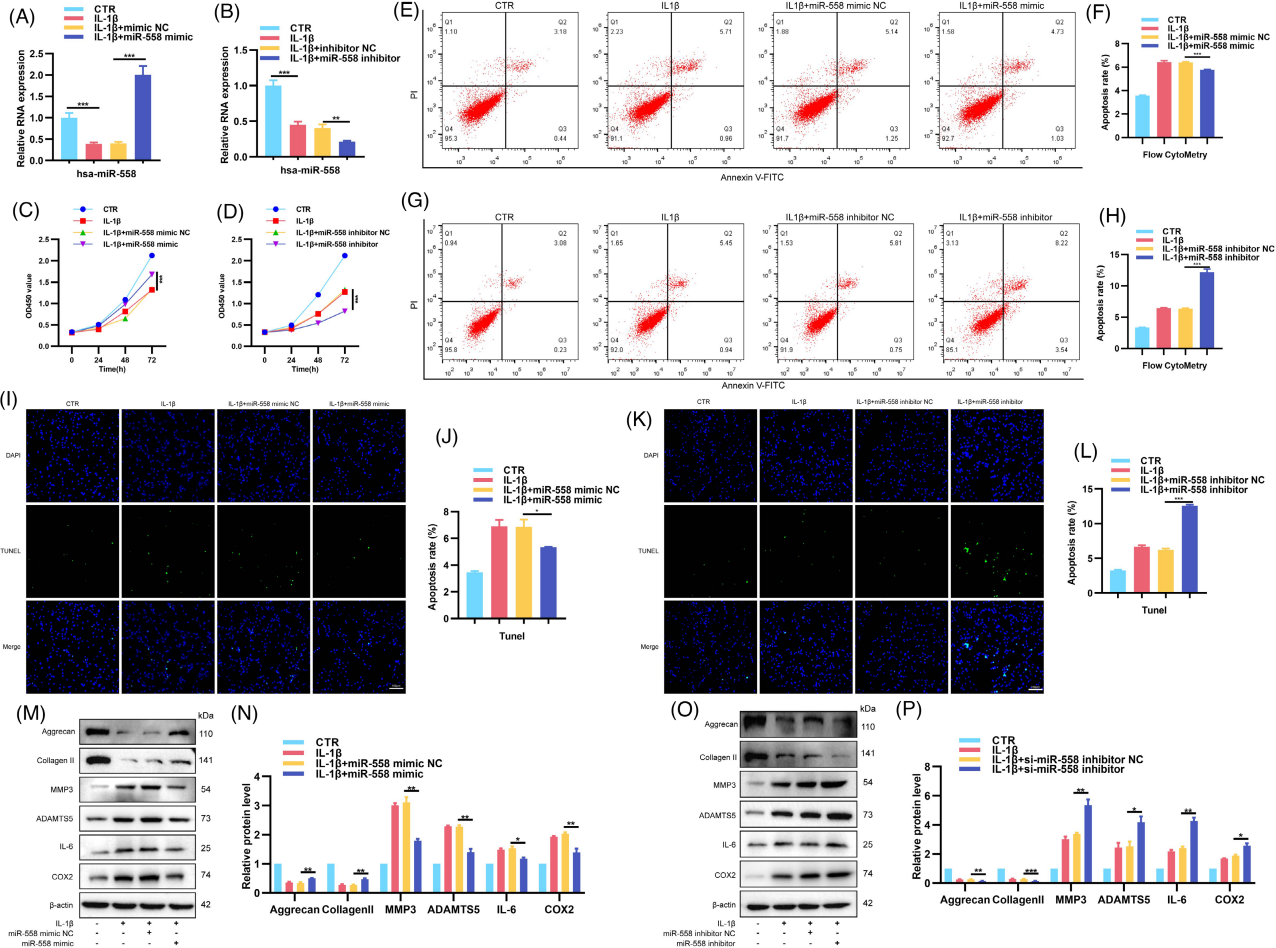
Roles of miR‐558 in the proliferation, apoptosis, ECM synthesis and degradation, and inflammatory factor release in NP cells. (A, B) NP cells treated with IL‐1β were transfected with miR‐558 mimic, inhibitor or NC, and levels of miR‐558 were detected by RT‐qPCR. (C, D) CCK8 assay examined proliferation of NP cells treated by IL‐1β with or without miR‐558 mimic or miR‐558 inhibitor. (E–L) Flow cytometry and TUNEL assay were used to detect NP cells apoptosis. (M–P) NP cells were transfected with miR‐558 mimic, inhibitor or NC before IL‐1β stimulation, and protein levels of aggrecan, collagen Ⅱ, MMP3, ADAMTS5, IL‐6 and COX2 were detected. *p < 0.05, **p < 0.01 and ***p < 0.001 vs. the indicated group. Statistical data were presented as mean ±SEM; FITC, fluorescein isothiocyanate; PI, propidium iodide; TUNEL, terminal dexynucleotidyl transferase (TdT)‐mediated dUTP nick‐end labelling; DAPI, 4′,6‐diamidino‐2‐phenylindole

As miR‐558 is the circ‐83756‐associated miRNA in NP cells, we subsequently explored whether miR‐558 participated in IVDD development. NP cells were transfected with miR‐558 mimics or inhibitors or their negative control, with or without IL‐1β. The RT‐qPCR results showed that IL‐1β treatment reduced miR‐558 levels in human NP cells, which could be reversed and aggravated by the miR‐558 mimic and miR‐558 inhibitor, respectively (Figure 4A,B). Functionally, CCK8 tests showed that miR‐558 mimic relieved, whilst miR‐558 inhibitor deteriorated the proliferation suppression of NP cells induced by IL‐1β (Figure 4C,D). Flow cytometry analysis and TUNEL staining showed that miR‐558 mimic reduced the rate of NP cells apoptosis induced by IL‐1β and miR‐558 inhibitor increased the apoptotic NP cells rate under IL‐1β stimulation (Figure 4E–L). Additionally, miR‐558 mimic resulted in elevated expressions of collagen II and aggrecan, whilst reduced levels of MMP3, ADAMTS5, IL‐6 and COX2 in the IVDD cellular model (Figure 4M,N). In contrast, the miR‐558 inhibitor led to increased expressions of MMP3, ADAMTS5, IL‐6 and COX2 but decreased expressions of collagen II and aggrecan in the same model (Figure 4O,P). These data suggested the regulatory function of miR‐558 on NP cells' biological processes.

### 2.5 Circ‐83756 regulated NP cells' biological function via binding to miR‐558

**FIGURE 5:**
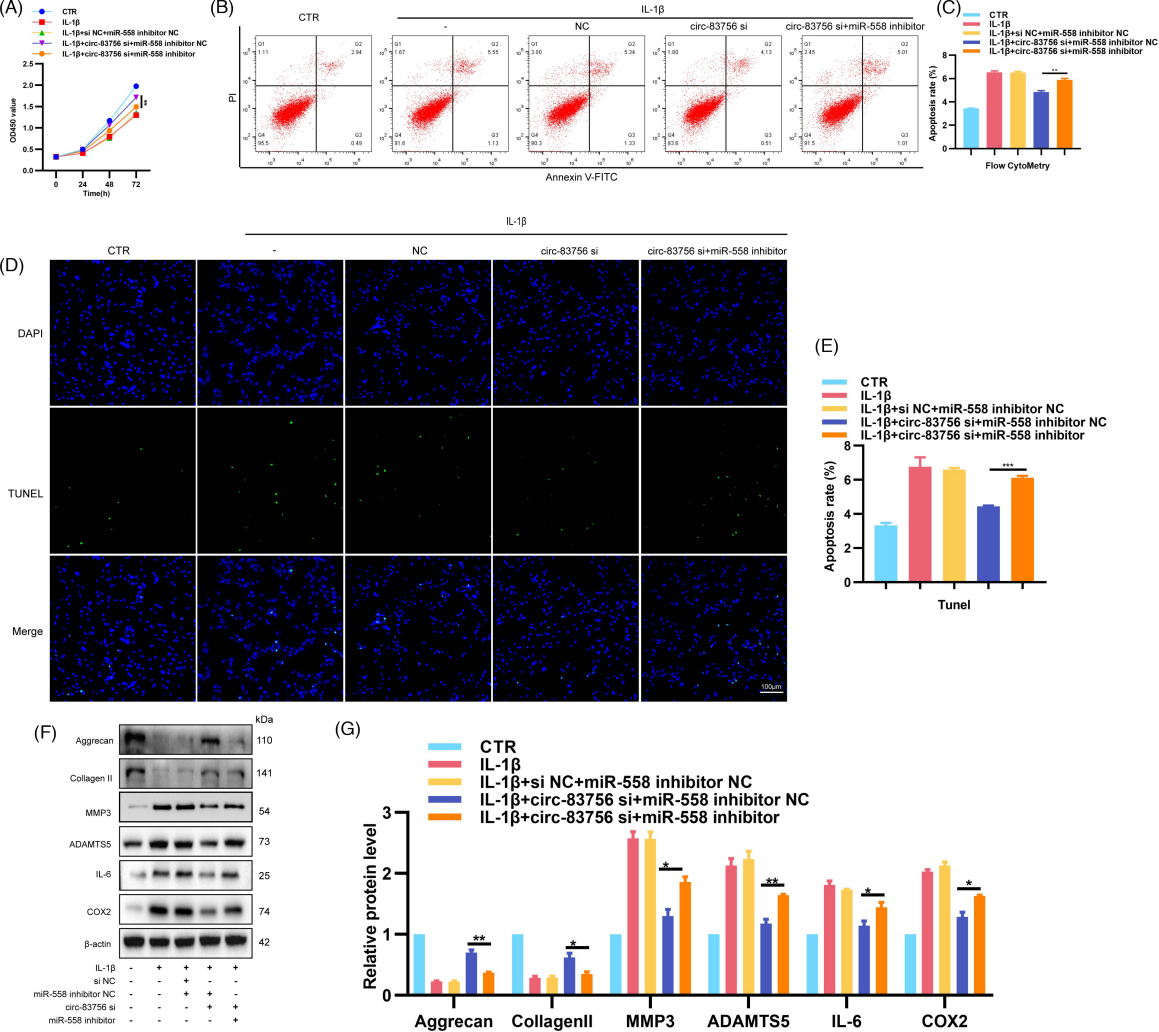
circ‐83756 played its roles in NP cells by sponging miR‐558. (A) Knockdown of miR‐558 expression reduced the circ‐83756‐si induced proliferation of NP cells. NP cells proliferation was examined by CCK8 assay. (B–E) Knockdown of miR‐558 expression resulted in more apoptotic NP cells than those observed with the inhibition of circ‐83756 alone. Apoptosis rates were determined with flow cytometry and TUNEL assay. (F, G) Expressions of aggrecan, collagen Ⅱ, MMP3, ADAMTS5, IL‐6 and COX2 in NP cells were detected by WB. Cells were co‐infected with circ‐83756 si and miR‐558 inhibitor or negative control. *p < 0.05, **p < 0.01 and ***p < 0.001 vs. the indicated group. Statistical data were presented as mean ±SEM; FITC, fluorescein isothiocyanate; PI, propidium iodide; TUNEL, terminal dexynucleotidyl transferase (TdT)‐mediated dUTP nick‐end labelling; DAPI, 4′,6‐diamidino‐2‐phenylindole

To verify the interaction effect between circ‐83756 and miR‐558 in IVDD, circ‐83756 siRNA (circ‐83756 si) and miR‐558 inhibitor were co‐transfected into NP cells. The CCK8 results indicated that the miR‐558 inhibitor could partially eliminate the promotion of NP cells proliferation induced by circ‐83756 si under IL‐1β stimulation (Figure 5A). The combined effects of circ‐83756 si and miR‐558 inhibitor on cell apoptosis were evaluated by flow cytometry and TUNEL staining. Compared with the control (CTR) group, IL‐1β increased the ratio of NP cells apoptosis, the silencing of circ‐83756 inhibited NP cells apoptosis, and the miR‐558 inhibitor could reverse the effects induced by circ‐83756 silencing (Figure 5B–E). Furthermore, in the IVDD cellular model, silencing of circ‐83756 increased the protein levels of aggrecan and collagen Ⅱ whilst reduced the protein levels of MMP3, ADAMTS5, IL‐6 and COX2, which were reversed by the addition of miR‐558 inhibitors (Figure 5F,G). Taken together, these data suggested that circ‐83756 regulated NP cells' biological function by sponging miR‐558.

### 2.6 TREM1 was the target gene of miR‐558

**FIGURE 6:**
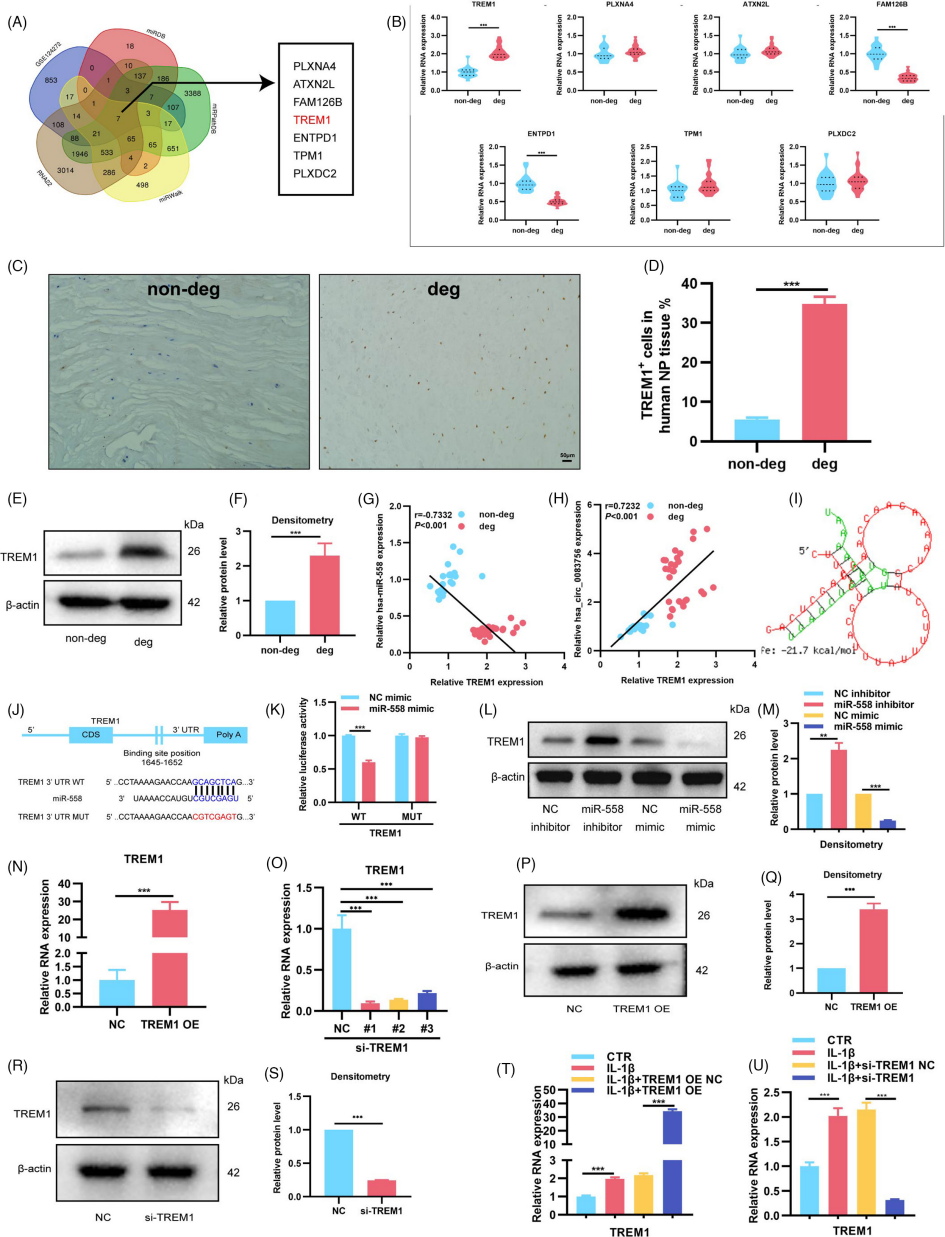
TREM1 was the target gene of miR‐558. (A) Venn diagram demonstrated the intersection of predicted potential target mRNAs of miR‐558 using the miRDB, miRPathDB, miRWalk and RNA22 databases, as well as the GSE124272 data set; there were seven candidate genes. (B) RT‐qPCR was used to detect the expressions of these seven candidate genes and TREM1 increased highest in degenerated NP tissues; *n* = 10 for non‐degenerated NP tissues and *n* = 14 for degenerated NP tissues. (C–F) IHC staining and WB showed that the expressions of TREM1 were upregulated in degenerated NP tissues. (G, H) Correlation analysis demonstrated that the expression of TREM1 was significantly positively correlated with circ‐83756 but negatively correlated with miR‐558 expression. (I) General structure diagram showing the binding sites of TREM1 to miR‐558 analysed by the bioinformatics programs and RNAhybrid. (J) Sequence alignment of human miR‐558 and the 3′‐UTR region of TREM1 mRNA. Bottom: mutations in the 3′‐UTR region of TREM1 to create mutant luciferase reporter constructs. (K) Luciferase reporter assay showed the luciferase activities of Luc‐TREM1 WT and Luc‐TREM1 MUT in T293 cells co‐transfected with miR‐NC or miR‐558 mimic. (L, M) WB showed that the expression of TREM1 was elevated by the miR‐558 inhibitor and suppressed by the miR‐558 mimic. (N, O) RT‐qPCR evaluated the expressions of TREM1 in NP cells transfected with TREM1 OE plasmid or three different TREM1 siRNAs. (P–S) WB showed that TREM1 OE can significantly upregulate the expression of TREM1, whereas si‐TREM1 decreases the expression of TREM1. (T, U) NP cells treated with IL‐1β were transfected with TREM1 OE or si‐TREM1, and TREM1 levels were detected by RT‐qPCR. **p < 0.01 and ***p < 0.001 vs. the indicated group. Statistical data are presented as mean ±SEM; IVDD, intervertebral disc degeneration; WT, wild type; MUT, mutant type

We used miRDB, miRPathDB, miRWalk, RNA22 and the GSE124272 database to predict the targeting genes of miR‐558. The venn diagram showed that there were seven potential genes, including TREM1, PLXNA4, ATXM2L, FAM126B, ENTPD1, TPM1 and PLXDC2 (Figure 6A). RT‐qPCR revealed that TREM1 was the most significantly increased gene in degenerated IVD specimens compared with non‐degenerated IVD specimens (Figure 6B). We further verified the expression patterns of TREM1 in IVD specimens using immunohistochemistry (IHC) and WB, which showed that the TREM1 protein levels were obviously upregulated in degenerated IVD NP tissues (Figure 6C–F and Figure S1). Pearson's correlation analysis identified a positive correlation between circ‐83756 and TREM1 and a negative correlation between miR‐558 and TREM1 (Figure 6G,H). The binding sites between miR‐558 and TREM1 were predicted by RNAhybrid^28^ and miRDB^29^ (Figure 6I,J). To confirm this finding, we constructed a dual‐luciferase reporter vector with the WT or MUT TREM1 3′‐untranslated region (UTR) possessing the putative miR‐558 target sites. Co‐transfection with miR‐558 mimics significantly decreased WT TREM1 reporter luciferase activity compared with the mimic control, whereas no significant change in luciferase activity was detected in the MUT 3′‐UTR of the TREM1 group (Figure 6K). WB was performed to further confirm the regulatory effect of miR‐558 on TREM1 protein levels, and the results demonstrated that the miR‐558 mimic decreased the expression of TREM1, whereas the miR‐558 inhibitor increased its expression (Figure 6L,M). Those results indicated that miR‐558 could directly bind the 3′‐UTR of TREM1 and negatively regulate its expression. Furthermore, to deeply investigate the effect of TREM1 on IVDD, we constructed TREM1 overexpression plasmid (TREM1 OE) and siRNAs to overexpress or silence TREM1 expression, respectively. The RT‐qPCR results confirmed that TREM1 OE significantly increased, whilst siRNAs obviously decreased TREM1 mRNA levels compared with their negative control (Figure 6N,O). As si‐TREM1 #1 exerted the best silencing effect, it was selected for the next experiments. WB studies further validated their overexpression or silencing effects (Figure 6P–S). In addition, the effects of TREM1 OE or si‐TREM1 on the expression of TREM1 were also confirmed in the human IVDD cellular model stimulated by IL‐1β (Figure 6T,U). Furthermore, TREM1 protein expression was upregulated after IL‐1β treatment (Figure S2).

### 2.7 circ‐83756 regulated IVDD by modulating the miR‐558/TREM1 axis

**FIGURE 7:**
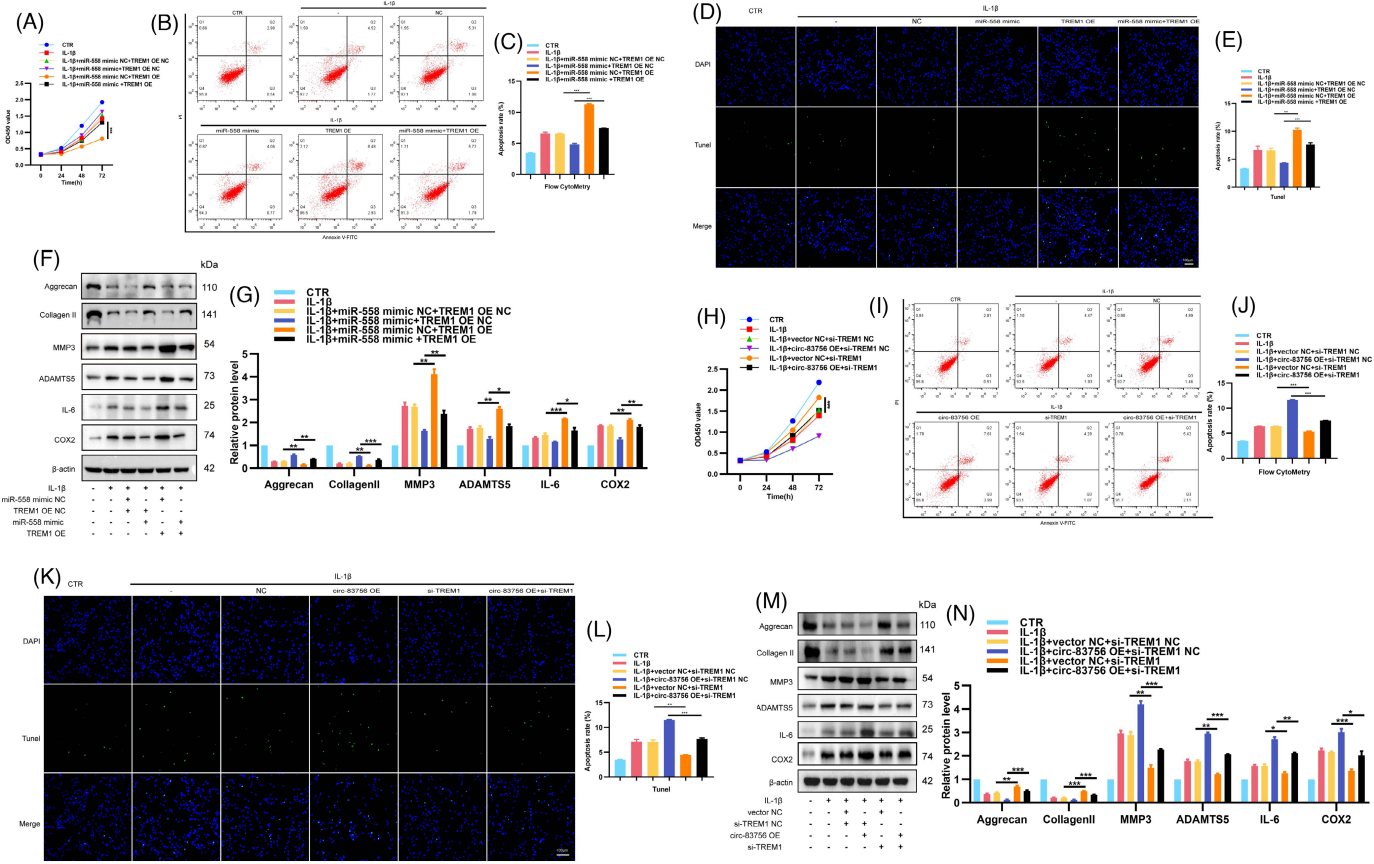
circ‐83756 regulated IVDD by modulating the miR‐558/TREM1 axis. (A) CCK8 assay detected the proliferation of NP cells co‐transfected with miR‐558 mimic and TREM1 OE. (B–E) Apoptosis rates were determined with flow cytometry and TUNEL assay. NP cells were co‐transfected with miR‐558 mimic and TREM1 OE. (F, G) The expressions of aggrecan, collagen Ⅱ, MMP3, ADAMTS5, IL‐6 and COX2 in NP cells were detected by WB. NP cells were co‐transfected with miR‐558 mimic and TREM1 OE. (H) The proliferation of NP cells co‐transfected with circ‐83756 OE and si‐TREM1 was detected by CCK8 assay. (I–L) Apoptosis rates were determined with flow cytometry and TUNEL assay. NP cells were co‐transfected with circ‐83756 OE and si‐TREM1. (M, N) The expressions of aggrecan, collagen Ⅱ, MMP3, ADAMTS5, IL‐6 and COX2 in NP cells were detected by WB. NP cells were co‐transfected with circ‐83756 OE and si‐TREM1. *p < 0.05, **p < 0.01 and ***p < 0.001 vs. the indicated group. Statistical data were presented as mean ±SEM; FITC, fluorescein isothiocyanate; PI, propidium iodide; TUNEL, terminal dexynucleotidyl transferase (TdT)‐mediated dUTP nick‐end labelling; DAPI, 4′,6‐diamidino‐2‐phenylindole

To verify whether the effects of circ‐83756 on IVDD were achieved through the miR‐558/TREM1 axis, we designed a set of rescue experiments. First, miR‐558 mimic and TREM1 OE or their negative control were co‐transfected into NP cells, respectively. In IL‐1β‐treated human NP cells, miR‐558 mimic upregulated NP cells proliferation, TREM1 OE downregulated NP cells proliferation, and TREM1 OE could partially attenuate the upregulation of NP cells proliferation induced by miR‐558 mimic (Figure 7A). Flow cytometry and TUNEL staining demonstrated that TREM1 OE elevated the NP cells apoptosis rate, which partially abolished the regulation of the miR‐558 mimic on NP cells apoptosis (Figure 7B–E). The WB and ELISA results indicated that miR‐558 mimic promoted ECM synthesis and inhibited ECM degradation and inflammatory factor release, whilst TREM1 overexpression impaired this protective effect (Figure 7F–G and Figure S3A). Second, we co‐transfected NP cells with circ‐83756 OE and si‐TREM1. CCK8 tests showed that si‐TREM1 increased the proliferation of NP cells, and si‐TREM1 could partially abolish the downregulation of circ‐83756 OE on NP cells proliferation (Figure 7H). Flow cytometry and TUNEL staining revealed that si‐TREM1 reduced the NP cells apoptosis rate, which partially ameliorated the pro‐apoptotic effect of circ‐83756 OE (Figure 7I–L). WB and ELISA also confirmed that the regulatory effect of circ‐83756 OE on ECM synthesis, degradation and inflammation factor release could be partially reversed by si‐TREM1 (Figure 7M,N and Figure S3B). Taken together, these data suggested that circ‐83756 regulated the process of IVDD by modulating the miR‐558/TREM1 axis.

### 2.8 Silencing circ‐83756 alleviated IVDD in a rat model

**FIGURE 8:**
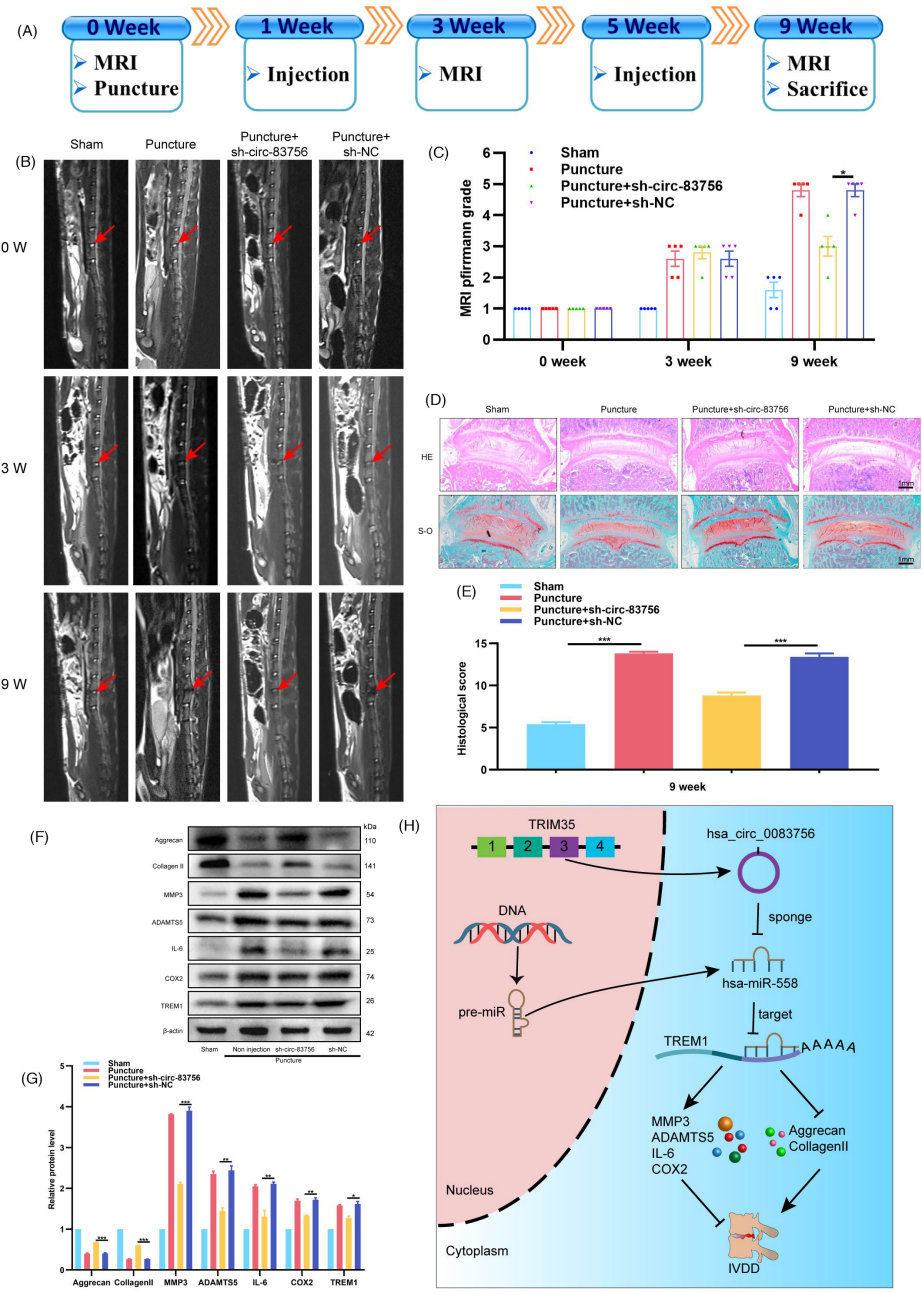
Silencing circ‐83756 alleviated rat IVDD in vivo. (A) Flow diagram of the experiments *in vivo*. (B) MRIs of the indicated groups were obtained at 0, 3 and 9 weeks after needle puncture (*n* = 5 in each group). (C) The degree of disc degeneration by MRI grade was significantly lower in the silencing circ‐83756 group than other groups. (D) H&E (top) and safranin‐O/fast green (bottom) staining of IVDs in the indicated groups at 9 weeks after needle puncture. (E) A significant decrease in intervertebral disc histological score was noted in the silencing circ‐83756 group compared with the other groups. (F, G) WB analysis determined the protein levels of aggrecan, collagen II, MMP3, ADAMTS5, IL‐6, COX2 and TREM1 in the rat NP tissues at 9 weeks. (H) Summary of the findings and schematic of the proposed mechanisms for circ‐83756. *p < 0.05, **p < 0.01 and ***p < 0.001 vs. the indicated group. Statistical data are presented as mean ± SEM

To confirm the effect of circ‐83756 *in vivo*, we established a rat IVDD model by needle puncture.^30^ Adeno‐associated virus (AAV) sh‐circ‐83756 or its negative control (NC) was injected into lumbar IVDs at weeks 1 and 5, following the experimental protocol (Figure 8A). Magnetic resonance imaging (MRI) was used to evaluate the severity of disc degeneration at 0, 3 and 9 week using the Pfirrmann classification.^31^ At the 9th week, the MRI degeneration scores of the IVDs were significantly lower in the AAV‐sh‐circ‐83756‐injected group than they were in the AAV sh‐NC injection group or IVD puncture group (Figure 8B,C). Then, HE and SO staining were used to assess the histomorphological changes in the IVD tissues^32^, ^33^: a clear framework with red‐stained NP, fibrous rings and cartilage plates in the sham group, and collapse of disc height, loss of NP tissue and decreased volume of proteoglycan matrix in the IVD puncture group. The injection of AAV‐sh‐circ‐83756 alleviated the loss of NP tissue and the destruction of disc structure (Figure 8D). A quantitative analysis indicated that the AAV‐sh‐circ‐83756‐injected group demonstrated significantly lower histological scores, whereas the AAV‐sh‐NC‐injected group had scores similar to those obtained for the IVD puncture group (Figure 8E). In addition, the injection of AAV‐sh‐circ‐83756 alleviated the degenerative changes of the IVDs, represented as decreased expressions of MMP3, ADAMTS5, IL‐6, COX2 and TREM1, and increased expressions of aggrecan and collagen II in the rat IVDD model (Figure 8F,G). Taken together, these results revealed the therapeutic role of circ‐83756 silencing in protecting IVD from degeneration *in vivo*. In conclusion, Circular RNA hsa_circ_0083756 promotes intervertebral disc degeneration by sponging miR‐558 and regulating TREM1 expression (Figure 8H).

## 3 DISCUSSION

Intervertebral disc degeneration is one of the main contributors to LBP and a leading cause of disability worldwide. Accumulating studies have demonstrated that circRNAs participate in the regulation of diverse diseases, including cancer,^34^ cardiovascular disease,^35^ immune diseases^36^ and osteoarthritis.^37^ Recently, circRNAs have also been identified to function as key regulators of IVDD.^38^, ^39^ However, the role and precise molecular mechanisms of circRNAs in IVDD remain elusive. In this study, we identified a novel circRNA named circ‐83756, which was upregulated in degenerative NP tissues and in an IVDD cell model. Next, we evaluated the function and mechanism of circ‐83756 in the regulation of IVDD. The results clarified that circ‐83756 contributed to the promotion of IVDD via upregulating TREM1 expression by sponging miR‐558. Furthermore, *in vivo* studies demonstrated that silencing of circ‐83756 could alleviate IVDD. Taken together, our data showed that circ‐83756 promotes IVDD via the miR‐558/TREM1 axis.

Previous studies suggested that circRNA participated in a variety of cellular processes, including proliferation, apoptosis, ECM synthesis and catabolism, and release of proinflammatory cytokine,^5^ which then influenced the physiological functions of IVD. For instance, circRNA‐CIDN could inhibit compression‐induced apoptosis and NP ECM degradation.^40^ circERCC2 ameliorated intervertebral disc degeneration by regulating mitophagy and apoptosis.^41^ Cui et al. reported that circ_001653 silencing promoted the proliferation and ECM synthesis of NP cells in IVDD.^42^ In the present study, we used our previous circRNA array analysis^24^ to intersect with the circRNA GSE67566 data set^43^ and found seven potential upregulated circRNAs. circ‐83756 was selected as the focus circRNA because it had the most consistent and largest difference in expression between the non‐degenerated and degenerated NP tissues. We then investigated the potential effects of circ‐83756 on the process of IVDD using an IL‐1β‐induced IVDD cell model.^6^ Gain‐of‐function and loss‐of‐function assays revealed that circ‐83756 inhibited NP cell proliferation, increased NP cell apoptosis, decreased collagen Ⅱ and aggrecan levels, as well as elevated MMP3, ADAMTS5, IL‐6 and COX2 levels. These findings suggested that circ‐83756 may play a vital role in the development of IVDD by influencing NP cell proliferation, apoptosis, ECM composition and proinflammatory cytokine release.

Mechanistically, circRNAs localizing in cytoplasm have been demonstrated to exert their functions by regulating the miRNA–mRNA axis.^44^ In this study, we found that circ‐83756 was localized in the cytoplasm of NP cells, indicating that circ‐83756 may regulate gene expression at the post‐transcriptional level. Based on the bioinformatics analysis, we found that circ‐83756 harbours the miR‐558 target sites that were validated by luciferase assay, FISH staining and RNA immunoprecipitation. Furthermore, we found that miR‐558 significantly decreased in degenerated NP tissues and was negatively regulated by circ‐83756 in the IL‐1β‐induced IVDD cellular model, suggesting that miR‐558 may participate in the process of IVDD mediated by circ‐83756. Our function study revealed that overexpression of miR‐558 induced NP cells proliferation, inhibited NP cells apoptosis, increased collagen Ⅱ and aggrecan levels. In addition, decreased MMP3, ADAMTS5, IL‐6 and COX2. Moreover, the results of rescue assays indicated that the *in vitro* protective action of circ‐83756 silencing was attenuated with the miR‐558 inhibitor. These findings suggest that circ‐83756 may exert its function in the process of IVDD by sponging to miR‐558, which is similar to the findings of previous studies.

Previous studies have confirmed that miRNA exerts its role by modulating its target gene expression,^45^, ^46^ inhibiting translation or promoting degradation. For example, miR‐98 can promote IVDD through the IL‐6/STAT3 signalling pathway,^47^ whereas miRNA‐558 promotes gastric cancer progression by attenuating Smad4‐mediated repression of heparinase expression.^48^ Our findings were similar to those of the above studies. In this study, bioinformatics predicted seven potential targets of miR‐558. Amongst all these potential targets, TREM1 was upregulated in degenerated NP tissues and could be regulated by both circ‐83756 and miR‐558 in NP cells. In addition, functional study and rescue experiments revealed that overexpression of TREM1 accelerated the development of IVDD, which could partly reduce the protective effect of miR‐558 mimic. Moreover, another rescue experiment showed that promotion of IVDD mediated by overexpression of circ‐83756 was significantly recuperated following TREM1 silencing. Taken together, the findings revealed that a circ‐83756/miR‐558/TREM1 axis exists in the development of IVDD.

To further investigate the effect of circ‐83756, we adopted an *in vivo* rat IVDD model by needle puncture to mimic the IVDD environment. The results showed that silencing of circ‐83756 alleviated the progression of IVDD, as shown by MRI assessment, histological evaluation and WB analysis. These results suggested that circ‐83756 may be a promising therapeutic target for IVDD.

Despite the encouraging findings, there are still some concerns that need to be addressed in the future. First, since patients with IVDD mainly complain of LBP or leg pain, the effect of circ‐83756 on pain management should be explored carefully. Second, because aberrant cartilage endplate and annulus fibrosus are also vital causes of IVDD, further investigations should comprehensively explore the involvement of circ‐83756 in the cartilage endplate and annulus fibrosus.

## 4 CONCLUSION

The present study demonstrated that circ‐83756 was significantly increased in degenerative NP tissues and cells. Mechanically, circ‐83756 promoted the development of IVDD by working as a ceRNA to sponge miR‐558, reinforcing the protein level of TREM1. This study provides a new reference for understanding the molecular pathophysiology mechanisms of IVDD and offers a potential therapeutic option for IVDD. However, more relevant mechanisms of action and treatment strategies need to be further explored.

## 5 MATERIALS AND METHODS

### 5.1 Human NP tissue collection and grading

Samples were collected under the ethics committee's approval, and informed consents were obtained before collection. All NP tissues were collected from patients undergoing spinal surgery in our hospital. Control non‐degenerated NP tissues were obtained from patients suffering from vertebral fracture or scoliosis, and degenerated NP tissues were collected from patients suffering from lumbar stenosis or disc herniation. MRI was performed for IVD degeneration assessment according to the method described by Pfirrmann et al.^31^ The clinical characteristics are listed in Table S1.

### 5.2 Isolation of NP cells and treatment

Human NP cells were isolated using a previously described method.^24^ Briefly, NP tissues were separated from surgical specimens, washed twice with sterile normal saline and cut into small pieces. Then, 0.25% trypsin and 0.2% collagenase Ⅱ (Sigma) were used to digest them for 1–2 h at 37°C. Next, the digestive solution was filtered with a 100 μM cell strainer and centrifuged at 1,500 rpm for 5 min. Following this, the NP cells were transferred and cultured in Dulbecco's Modified Eagle's Medium (DMEM) with 10% foetal bovine serum (Invitrogen), as well as 1% penicillin/streptomycin (Invitrogen) in a 37°C, 5% CO_2_ incubator. The medium was changed every 2–3 days. The second to fourth passages were used for experiments. To establish a stable IVDD cell model, the cells were treated with IL‐1β (Peprotech) for 24 h.

### 5.3 Data extraction and analysis

CircRNA sequences and annotations were obtained from circBase (http://www.circbase.org/). The miRNA sequence was obtained from miRbase (http://www.mirbase.org/). The GSE67566 database was downloaded from the GEO database (http://www.ncbi.nlm.nih.gov/geo). Algorithms, including circBank (http://www.circbank.cn/) and CircInteractome (https://circinteractome.nia.nih.gov/), were used to detect the potential miRNAs binding to individual circRNA. The miRDB (http://mirdb.org/), miRPathDB (https://mpd.bioinf.uni‐sb.de/), miRWalk (http://mirwalk.umm.uni‐heidelberg.de/), RNA22 (https://cm.jefferson.edu/rna22/), and GSE124272 databases were used to predict the potential targets of individual miRNAs.

### 5.4 RT‐qPCR

Total RNA was extracted using TRIzol reagent (Thermo Fisher Scientific), then applied for cDNA synthesis using SuperScript cDNA synthesis kits (YEASEN) (with random primers for circular RNAs). Following this, cDNA was used for qPCR with qPCR Master Mix (YEASEN). The result was calculated using the 2^−ΔΔCt^ method. U6 was used as control for miRNA, and β‐actin was used as control for circRNA and mRNA. The primers used in qPCR are listed in Table S2.

### 5.5 Western blot

After treatment, cells were washed twice with cold phosphate‐buffered saline (PBS) and lysed with prepared radioimmunoprecipitation assay (RIPA) buffer containing 1% PMSF (Fude) on ice for 15–30 min. Then, the lysate was collected into a new tube and concentrated at ~14,000 ×*g* for 15 min. The protein concentration was determined using a bicinchoninic acid (BCA) kit (YEASEN, and the proteins were separated by 8%–10% sodium dodecyl sulphate–polyacrylamide gel electrophoresis (SDS‐PAGE). Subsequently, the proteins were transferred onto a PVDF membrane (Millipore, USA) and blocked with 5% bovine serum albumin (BSA) at room temperature for 2 h. The membrane was then incubated with specific primary antibodies against aggrecan (Abcam, ab3773, 1:200), collagen II (Abcam, ab188570, 1:2000), MMP3 (Abcam, ab53015, 1:1000), ATAMTS5 (Abcam, ab41037, 1:250), IL‐6 (Abcam, ab9324, 1:1000), COX2 (Abcam, ab15191, 1:1000), TREM1 (Abcam, ab200729, 1:1000), β‐actin (Servicebio, GB11001, 1:2000). The membrane was then incubated with the horseradish peroxidase (HRP)‐conjugated secondary antibody after washing the membranes three times using TBST. Next, the membrane was detected using an ECL reagent (Thermo Fisher Scientific) at room temperature. The grey intensity of the proteins was measured using ImageJ software (NIH, USA).

### 5.6 Plasmid construction and NP cell transfection

For circRNA‐overexpressing vectors, the full‐length of circ‐83756 was cloned and constructed into pK25ssAAV‐ciR (Geneseed). The specific small interfering RNA (siRNA) of circ‐83756 and its negative control were synthesized by Hanbio Technology. The full‐length sequences of TREM1 CDS were amplified using PCR methods and constructed into a pCDNA3.1(+) vector for overexpression. Silencing of TRME1 was achieved using siRNA obtained from RIBOBIO Technology. Lipofectamine RNAiMAX reagent or Lipofectamine 3000 (Thermo Fisher Scientific) was applied for NP cell transfection according to the manufacturer's instructions.

### 5.7 Luciferase reporter assay

The complementary relationship between circ‐83756 and miR‐558 was predicted with CircInteractome (https://circinteractome.nia.nih.gov/). miRDB (http://mirdb.org/) was used to predict the binding sites of miR‐558 and TREM1 mRNA 3′UTR. The putative miR‐558 target‐binding sequences and mutant sequences in circ‐83756 were synthesized and constructed into the pmirGLO promoter vector (Promega). WT pmirGLO‐circ‐83756 or MUT pmirGLO‐circ‐83756 reporter plasmid and miR‐558 mimic (or mimic NC) were co‐transfected into T293 cells using Lipofectamine 3000 reagents (Invitrogen). To confirm the direct interaction of miR‐558 and TREM1, TREM1 WT or mutated reporter plasmid for the luciferase assay was also constructed and co‐transfected into T293 cells with lipofectamine 3000 reagents. The luciferase activity was detected with the Dual‐Luciferase Reporter Assay System (Promega) under the manufacturer's instructions.

### 5.8 RNA FISH

RNA FISH was performed to verify the subcellular localizations of circ‐83756 and miR‐558. CY5‐labeled circ‐83756 probes and 6‐FAM‐labeled miR‐558 probes were designed and synthesized by Bersinbio (Guangzhou, China). The experiment was performed using a FISH Kit (Bersinbio) according to the manufacturer's guidelines. Briefly, NP cells were seeded into six‐well culture plates and cultured until confluence reached about 70%. Then, the cells were fixed with 4% paraformaldehyde and permeabilized with 0.1% Triton X‐100 at room temperature. The cells were hybridized with a hybridization solution containing a probe at 37°C overnight. Following this, the cells were washed using saline sodium citrate (SSC) solution and stained with DAPI for 10 min without light. The images were obtained using a fluorescence microscope (Zeiss).

### 5.9 RIP assay

The Ago2‐RIP experiments were performed using an RNA‐Binding Protein Immunoprecipitation Kit (Millipore, USA). Briefly, NP cells were lysed in RNA lysis buffer and then added to the magnetic beads. The magnetic beads were conjugated to human anti‐Ago2 antibody or control anti‐IgG (Millipore) antibody in RIP immunoprecipitation buffer for 6 h at 4°C. Next, the samples were incubated with Proteinase K; immunoprecipitated RNA was then isolated and collected. Furthermore, purified RNAs were detected and analysed by RT‐qPCR to demonstrate the enrichment of circ‐83756 and miR‐558.

### 5.10 CCK8

Cell viability detection was performed using a CCK8 assay (YEASEN). Briefly, about 5 × 10^3^ of NP cells were seeded into each well of 96‐well plates. After the treatment, 10 μl of CCK8 agent was added to each cell. After 1.5 h of incubation at 37℃, the absorbance was measured with a microplate reader (Tecan, Spectra Fluor Plus) at 450 nm.

### 5.11 TUNEL staining

Transferase dUTP nick‐end labelling staining was performed to examine cell apoptosis. In brief, NP cells were fixed with 4% paraformaldehyde for 1 h and then cultured with 0.5% TritonX‐100 in PBS for 10 min. After washing with PBS three times, the cells were incubated following the instructions of the apoptosis detection kit (Servicebio) and stained with 4′,6‐diamidino‐2‐phenylindole (DAPI). Apoptosis was observed and photographed under a fluorescence microscope (Leica DMI8, Wetzlar, Germany).

### 5.12 Flow cytometric analysis

Nucleus pulposus cells were collected and detected using an Annexin V‐FITC Apoptosis Detection Kit (YEASEN) according to the manufacturer's instructions. Briefly, after washing, NP cells were resuspended and stained with Annexin V‐FITC and PI. Then, apoptotic cells were analysed using a flow cytometry system (Beckman Coulter).

### 5.13 Animal study

All animal studies were performed in accordance with the protocols approved by the institutional animal care and use committee of Sun Yat‐Sen University. Twenty male Sprague–Dawley rats were purchased from the laboratory animal centre of Sun Yat‐Sen University. An anterior disc‐puncture IVDD animal model was established according to our previous study. The L4/5 IVD was exposed and punctured as described previously.^30^ Rats were divided into the four following groups: a sham surgery group, a needle puncture group, a needle puncture +AVV sh‐circ‐83756 injection group and a needle puncture +AVV sh‐NC injection group. In the AVV sh‐circ‐83756 injection group or control NC injection group, 2 μl of the vectors were slowly injected into the punctured IVD through an anterior approach at 1 and 5 weeks after first surgery. Lumbar MRI examinations were performed at 0, 3 and 9 weeks after the first surgery. The Pfirrmann^31^ classification was used to assess the degree of IVD degeneration. After euthanasia, the L4/5 IVDs of the rats were excised together with adjacent vertebrae. The samples were cut into sections and stained with haematoxylin–eosin (H&E) and Safranin‐O. WB was used to evaluate the expression of cytokine and ECM composition‐related protein. The grading score of histological staining was made per the criteria established by Masuda et al.^32^ and Mao et al.^33^

### 5.14 Statistical analysis

All quantitative data are presented as the mean ±SEM. Statistical analyses were performed using GraphPad Prism version 8.0 (GraphPad Software Inc.). A one‐way analysis of variance (ANOVA) or Student's *t*‐test was used to analyse the differences between groups. Nonparametric test was taken to compare the difference between puncture +sh‐circ‐83756 group and puncture +sh‐NC group. The relationship between the expression of miR‐558 and circ‐83756 and between miR‐558 and TREM1 were explored by Pearson's correlation coefficient. For all statistical analyses, *p* < 0.05 was considered statistically different between groups.

## CONFLICT OF INTEREST

The authors declare no conflicts of interest in relation to this article.

## AUTHOR CONTRIBUTION

Xianfa Du, Hua Wang and Zhaomin Zheng designed the study. Xianfa Du, Shunlun Chen and Haitao Cui performed the experiments. Yuming Huang, Jianru Wang, Hui Liu, Zemin Li and Chunxiang Liang collected the clinical samples. Xianfa Du, Hua Wang contributed to the data analysis and manuscript draft. Zhaomin Zheng contributed to data analysis and corrected the manuscript. All authors read and approved the final manuscript.

## Supporting information

App S1

## Data Availability

The data sets used and/or analysed during the current study are available from the corresponding author on reasonable request.
