# Supplementary material for: Circular RNA hsa_circ_0083756 promotes intervertebral disc degeneration by sponging miR‐558 and regulating TREM1 expression
Source: Cell Prolif. 2022 Feb 21;55(4):e13205. doi: 10.1111/cpr.13205 (PMC9055908; doi:10.1111/cpr.13205)
Supplement: Supplementary file 1 — App S1 [file CPR-55-e13205-s001.docx]

**Appendix S1**

**Circular RNA hsa_circ_0083756 promotes intervertebral disc degeneration by sponging miR-558 and regulating TREM1 expression**

**Supplement material**

**Table S1 Patients information**

| Number | Age（y） | Gender | Pfirrmann grade | Level |
| --- | --- | --- | --- | --- |
| non-deg 1 | 12 | Male | G1 | L1/2 |
| non-deg 2 | 18 | Male | G2 | L4/5 |
| non-deg 3 | 3 | Male | G1 | L2/3 |
| non-deg 4 | 25 | Male | G2 | L4/5 |
| non-deg 5 | 31 | Male | G2 | L4/5 |
| non-deg 6 | 3 | Female | G1 | T11/12 |
| non-deg 7 | 12 | Male | G1 | T11/12 |
| non-deg 8 | 13 | Female | G1 | T5/6 |
| non-deg 9 | 32 | Male | G2 | L2/3 |
| non-deg 10 | 6 | Female | G1 | T8/9 |
| deg 1 | 32 | Female | G4 | L4/5 |
| deg 2 | 64 | Female | G5 | L3/4 |
| deg 3 | 49 | Female | G4 | L4/5 |
| deg 4 | 56 | Female | G4 | L4/5 |
| deg 5 | 69 | Female | G4 | L4/5 |
| deg 6 | 56 | Female | G5 | L4/5 |
| deg 7 | 48 | Male | G4 | L4/5 |
| deg 8 | 57 | Male | G4 | L4/5 |
| deg 9 | 48 | Female | G4 | L5/S1 |
| deg 10 | 66 | Female | G5 | L4/5 |
| deg 11 | 41 | Male | G5 | L4/5 |
| deg 12 | 60 | Female | G5 | L4/5 |
| deg 13 | 51 | Female | G4 | L4/5 |
| deg 14 | 57 | Male | G4 | L4/5 |

**Table S2 Primers sequence**

| Primers for qPCR | | |
| --- | --- | --- |
| hsa_circ_0083756 | F | CGACGTTTCTTTTCTCATGGTG |
|  | R | AGTTGCTTCTGCCTTGTCTC |
| TRIM35 | F | TGAAGGAGGACGACGTTTCTT |
|  | R | GCCCAGGTACTTGCAGACATC |
| hsa-miR-558 | F | GCGCGTGAGCTGCTGTAC |
|  | R | AGTGCAGGGTCCGAGGTATT |
| TREM1 | F | GAACTCCGAGCTGCAACTAAA |
|  | R | TCTAGCGTGTATCACATTTCAC |
| β-actin | F | CCTGGCACCCAGCACAAT |
|  | R | GGGCCGGACTCGTCATAC |
| U6 | F | CTCGCTTCGGCAGCACA |
|  | R | AACGCTTCACGAATTTGCGT |

**Fig S1**

**Fig. S1 The expression of TREM1 in clinical IVD samples**


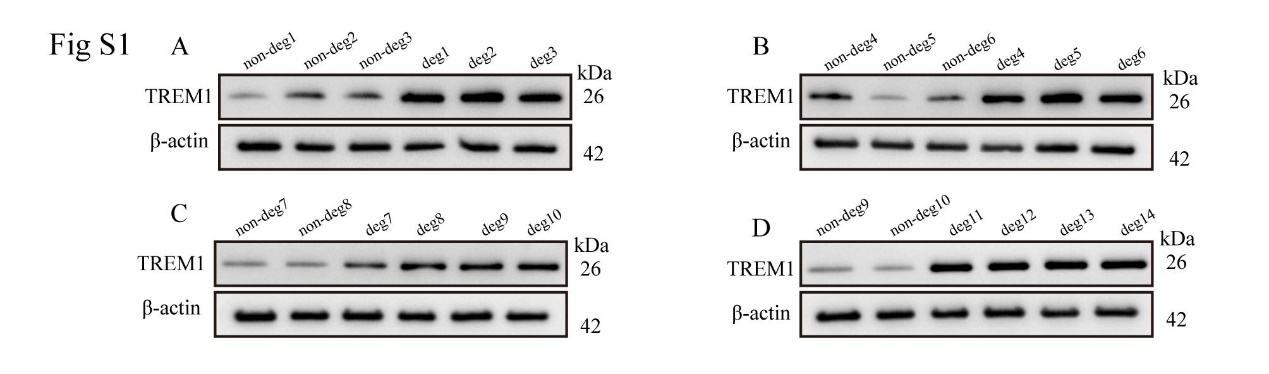


**(A-D)** The protein level of TREM1 in clinical samples was detected by WB. non-deg: non-degenerated; deg: degenerated

**Fig S2**


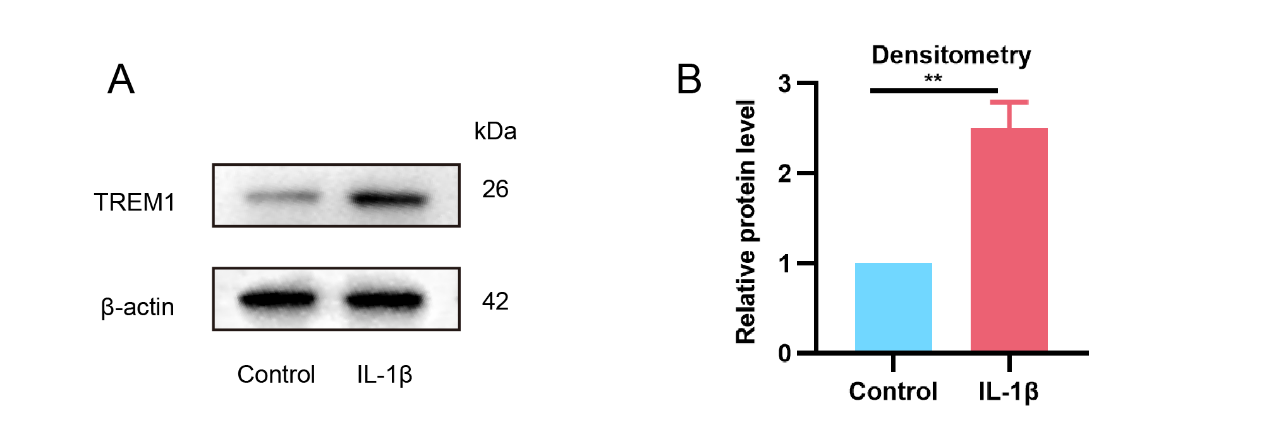
**Fig. S2 The protein level change of TREM1 after NP cells treated with IL-1β**

**(A-B)** Western blot and densitometric analysis showed TREM1 protein expression was upregulated after IL-1β treatment in NP cells. **p < 0.01 versus the indicated group. Statistical data were presented as mean ± SEM;

**Fig S3**

**Fig. S3 The supernate level of IL-6 in NP cells in the rescue experiments**


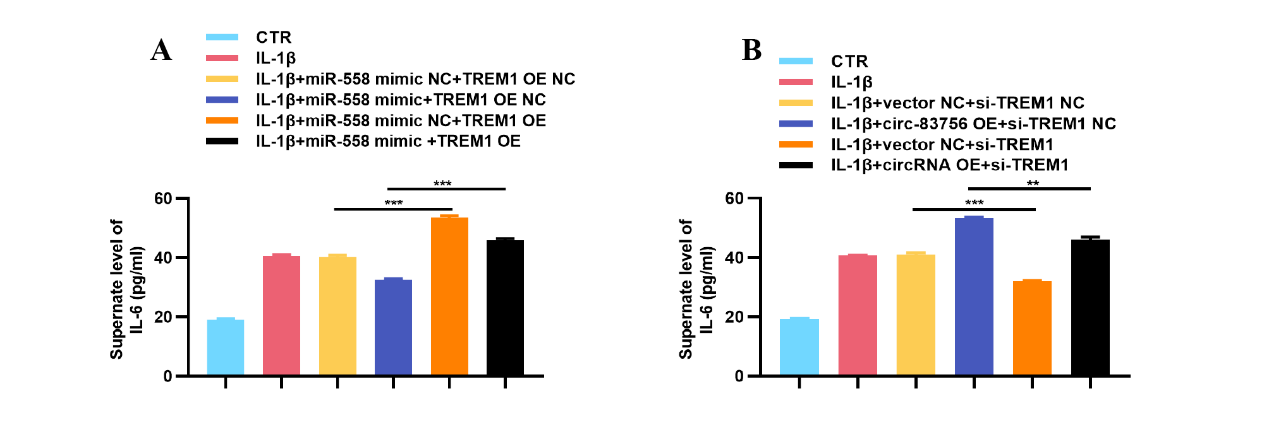


**A** The supernate level of IL-6 in NP cells were detected by ELISA. NP cells were co-transfected with miR-558 mimic and TREM1 OE. **B** The supernate level of IL-6 in NP cells were detected by ELISA. NP cells were co-transfected with circ-83756 OE and si-TREM1. *p < 0.05, **p < 0.01, and **p < 0.001 versus the indicated group. Statistical data were presented as mean ± SEM;
